# SIRT1 overexpression is an independent prognosticator for patients with esophageal squamous cell carcinoma

**DOI:** 10.1186/s13019-018-0718-5

**Published:** 2018-04-10

**Authors:** Ming-Chun Ma, Tai-Jan Chiu, Hung-I Lu, Wan-Ting Huang, Chien-Ming Lo, Wan-Yu Tien, Ya-Chun Lan, Yen-Yang Chen, Chang-Han Chen, Shau-Hsuan Li

**Affiliations:** 1 Department of Hematology-Oncology, Kaohsiung Chang Gung Memorial Hospital and Chang Gung University College of Medicine, 123 Ta-Pei Road, Niaosong Dist, Kaohsiung, Taiwan, Republic of China; 2 Department of Thoracic & Cardiovascular Surgery, Kaohsiung Chang Gung Memorial Hospital and Chang Gung University College of Medicine, Kaohsiung, Taiwan, Republic of China; 3 Department of Pathology, Kaohsiung Chang Gung Memorial Hospital and Chang Gung University College of Medicine, Kaohsiung, Taiwan, Republic of China; 4 Institute for Translational Research in Biomedicine, Kaohsiung Chang Gung Memorial Hospital, Kaohsiung, Taiwan, Republic of China; 5 Department of Applied Chemistry, and Graduate Institute of Biomedicine and Biomedical Technology, National Chi Nan University, Nantou, Taiwan, Republic of China; 6 Center for Infectious Disease and Cancer Research, Kaohsiung Medical University, Kaohsiung, Taiwan, Republic of China

**Keywords:** Esophageal squamous cell carcinoma, SIRT1, Overexpression

## Abstract

**Background:**

Sirtuin 1 (SIRT1) regulates DNA repair and metabolism by deacetylating target proteins. SIRT1 may be oncogenic because its overexpression has been detected in many cancers. The aim of the present study was to clarify the prognostic role of SIRT1 in patients with esophageal squamous cell carcinoma (ESCC) and evaluate the effect of SIRT1 inhibitor in vitro.

**Methods:**

The expression of SIRT1 was evaluated immunohistochemically in 155 surgically resected ESCC and the staining results were evaluated semiquantitatively by the Immunoreactive Scoring System. The clinical features and treatment outcome were analyzed. The effect of SIRT1 inhibitor, SIRT 1 inhibitor IV, (S)-35, was investigated in vitro on ESCC cell lines.

**Results:**

The expression of SIRT1 on ESCC did not correlate with age, gender, tumor location, stage, T classification, N classification, surgical margin or histology. Univariate analysis showed that SIRT1 overexpression was associated with inferior overall survival (*P* = 0.004) and disease-free survival (*P* = 0.004). In multivariate comparison, SIRT1 overexpression remained independently associated with worse overall survival (*P* = 0.009, hazard ratio = 1.776) and disease-free survival (*P* = 0.017, hazard ratio = 1.642). In cell lines, SIRT1 inhibitor inhibited ESCC growth.

**Conclusions:**

Our study suggests that SIRT1 overexpression is an independent prognosticator for patients with ESCC and the SIRT1 inhibitor suppressed cell proliferation of ESCC cell lines. Our findings suggest that inhibition of SIRT1 signaling may be a promising novel target for ESCC.

## Background

The standard therapy for patients with esophageal squamous cell carcinoma (ESCC) is surgery and concurrent chemoradiothrapy [1–3]. Much improvement was achieved during the past decades. However, the tumor recurs despite of extensive surgery, and the prognosis was still unsatisfactory [1, 4, 5]. Identification of prognostic biomarkers for ESCC is crucial for clinicians to make risk-adapted treatment plans and the potential for novel target therapy.

Mammalian sirtuins deacetylases consist of seven family members (SIRT1–7) that have been shown to be critical regulators of cell signaling pathways [6, 7]. SIRT1 is a NAD + −dependent deacetylase that plays important roles in many biological processes, including stress response, apoptosis, cellular metabolism, adaptation to calorie restriction, aging, and tumorigenesis.

Because some members of the various classes of histone deacetylases (HDACs) have been shown to be overexpressed in diverse cancers, current views suggest that perturbed acetylation patterns on proteins may contribute to cellular transformation and tumor progression [6, 8, 9]. SIRT1 can activate stress defense and DNA repair mechanisms, and therefore aids in the preservation of genomic integrity [10, 11]. SIRT1 also functions in the regulation of metabolism and maintaining the integrity of the genome, and thus has been described as a potential tumor suppressor [11]. For example, both breast cancer and hepatocellular carcinoma exhibit reduced SIRT1 levels compared with normal tissues [12]. Wang et al. [12] demonstrated that Sirt1(+/−); p53(+/−) mice develop tumors in multiple tissues, whereas activation of SIRT1 by resveratrol treatment reduces tumorigenesis, and thus suggested that SIRT1 may act as a tumor suppressor through its role in DNA damage response and genome integrity. Previous study [11] also showed that SIRT1 activity is required for suppressing survivin transcription, and reduction of survivin via SIRT1 activity may play an important role in breast cancer susceptibility gene 1 (BRCA1)-associated mammary tumor formation. Conversely, other studies showed that overexpression of SIRT1 caused the suppression of DNA damage repair proteins and factors involved in tumor suppression, and thus led to increased tumor growth and cell survival [11, 13]. Previous studies [11] revealed that SIRT1-mediated deacetylation suppresses the functions of several tumor suppressors, including p53, p73, and hypermethylated in cancer 1 (HIC1). Upregulation of SIRT1 has been reported in various human malignancies including prostate cancer, breast cancer, lung cancer, lymphoma, leukemia, soft tissue sarcomas, colon cancer, and gastric cancer [11, 14–19]. In recent years, a number of inhibitors have been discovered and characterized. This raises the possibility that SIRT1 inhibition might suppress cancer cell proliferation [11]. However, the role of SIRT1 in ESCC remains largely undefined. Therefore, we conducted the present study to evaluate the prognostic significance of SIRT1 in patients with ESCC by immunohistochemistry and investigate the effect of SIRT1 inhibitor in vitro.

## Methods

### Patient population

Patients with ESCC who received surgical resection at Kaohsiung Chang Gung Memorial Hospital were reviewed retrospectively. This study was approved by the Institutional Review Board of Chang Gung Memorial Hospital. The approval number of this project was 201800339B0. Patients with second malignancy and who receiving chemotherapy and/or radiotherapy before surgery were excluded. We identified 155 patients with available paraffin blocks and follow-up. Patients underwent a radical esophagectomy with cervical esophagogastric anastomosis (McKeown procedure) or an Ivor Lewis esophagectomy with intrathoracic anastomosis, reconstruction of the digestive tract with gastric tube, and pylorus drainage procedures. Two-field lymph node dissection was performed in all patients. The pathologic TNM stage was determined according to the 7th American Joint Committee on Cancer (AJCC) staging system. After surgery, patients were followed at 3-month intervals for 2 years, 6-month intervals up to year 5, and annually thereafter. Disease-free survival (DFS) was calculated from the time of operation to the recurrence or death from any cause without evidence of recurrence. Overall survival (OS) was calculated from the time of operation to death as a result of all causes.

### Immunohistochemistry

Immunohistochemistry was used to evaluate the expression of SIRT1. Formalin-fixed and paraffin-embedded 4-μm thick tumor tissue slices were dewaxed and rehydrated before antigen retrieval. The microwave antigen retrieval method was then utilized, and the slides were immersed in EDTA antigen retrieval solution (pH 9.0) for 15 min. Subsequently, we added 3% hydrogen peroxide to the slides to inhibit endogenous peroxidase activity. Subsequently, SIRT1 (1:150; Abcam, Cambridge, UK) was applied to the sections that were later incubated at 4 °C overnight. On the second day, biotinylated antibody and streptavidinperoxidase reagent were successively applied for 15 min each at 37 °C. Finally, 3,3′-diaminobenzidine tetrahydrochloride (DAB) was used for visualization, and hematoxylin was added as a counterstain. The positive controls were human non-small cell lung cancer tissues expressing SIRT1. Sections that were incubated with PBS instead of primary antibodies were used as negative controls. Both the positive and negative controls were used to evaluate the reliability of staining and exclude nonspecific reactions. The expression level of SIRT1 protein was calculated utilizing a semiquantitative scoring system. The staining score was classified as 0 (negative staining), 1 (weak staining), 2 (moderate staining) and 3 (strong staining). The quantity score, which represented the percentage of cancer cells that were positively stained, was calculated as follows: 0 (0–5%), 1 (6–25%), 2 (26–50%), 3 (51–75%), and 4 (≥76%). By multiplying the staining score by the quantity score of each slide, the final semiquantitative score was obtained (ranging from 0 to 12). Scores that ranged from 4 to 12 were considered to represent overexpression.

### Cell culture and 3-(4.5-dimethylthiazol-2-yl)-2,5-diphenyltetrazolium bromide assay

Human esophageal squamous cell carcinoma cell line KYSE 270 and KYSE 70 were obtained from the European Collection of Authenticated Cell Cultures (ECACC). The KYSE 270 cell line was maintained in RPMI 1640 (Invitrogen, Carlsbad, CA) and Ham’s F12 (Nissui Pharmaceutical, Tokyo, Japan) mixed (1:1) medium containing 2% fetal bovine serum. The KYSE 70 cell line was maintained in RPMI 1640 medium (Invitrogen, Carlsbad, CA) medium containing 10% fetal bovine serum. To test the effects of cell proliferation of SIRT1 inhibitor, SIRT 1 inhibitor IV, (S)-35 (Calbiochem, Merck Millipore, Darmstadt, Germany), cells were plated into 96-well, flat bottomed plates at 3 × 10^3^ cells per 100 mL per well in the recommended medium containing 10% fetal bovine serum. After overnight incubation, triplicate wells were treated with different concentrations of SIRT1 inhibitor (0, 5, 10 and 20 μM) for 24 h. The relative percentages of metabolically active cells compared with untreated controls were then determined on the basis of mitochondrial conversion of 3- (4.5-dimethylthiazol-2-yl)-2,5-diphenyltetrazolium bromide (MTT) to formazine. In brief, after incubation, 10 mL of MTT (Sigma, St Louis, MO) solution (5 mg/mL) was added to each well for 3 h, and the medium was then replaced with 150 mL of dimethylsulfoxide per well. Results were assessed in a 96-well format plate reader by measuring the absorbance at a wavelength of 540 nm using a Titertek Multiscan (Thermo, Vantaa, Finland).

### Statistical analysis

The SPSS software package (18.0; SPSS, Chicago, IL, USA) was used for statistical analysis. Correlations among SIRT1 and various clinicopathologic characteristics were compared using the chi-square test. Survival curves were constructed using the Kaplan-Meier method, and the significance of differences in the survival of subgroups was examined with the log rank test. Independent prognostic factors were determined by multivariate Cox regression analysis. *P* values less than 0.05 were considered significant.

## Results

### Patient characteristics

The median age for the 155 patients (150 men and 5 women) was 55 years (range, 29–77). The 7th AJCC stages of 155 patients with ESCC were stage I in 44 patients, stage II in 68, stage III in 39, and stage IV in 4 (Table 1). The histologic grading was grade 1 in 16 patients, grade 2 in 108, and grade 3 in 31. The tumor locations were upper esophagus in 22 patients, middle in 60, and lower in 73. At the time of analysis, the median periods of follow-up were 65.8 months (range, 50.4~ 238 months) for the 50 survivors and 37.6 months (range, 0.9~ 238 months) for all 155 patients. The 3-year OS and DFS rates for these 155 patients were 52% and 43%, respectively. The 5-year OS and DFS rates for these 155 patients were 44% and 37%, respectively.

**Table 1:** Characteristics of 155 patients with esophageal squamous cell carcinoma receiving esophagectomy

Age	median	55
	mean	56.2
	range	29~ 77
Sex	male	150 (97%)
	female	5 (3%)
Primary tumor location	Upper	22 (14%)
	Middle	60 (39%)
	Lower	73 (47%)
T classification	T1	48 (31%)
	T2	31 (20%)
	T3	61 (39%)
	T4	15 (10%)
N classification	N0	106 (68%)
	N1	30 (19%)
	N2	13 (9%)
	N3	6 (4%)
7^th^ AJCC Stage	IA	7 (5%)
	IB	37 (24%)
	IIA	25 (16%)
	IIB	43 (28%)
	IIIA	15 (9%)
	IIIB	6 (4%)
	IIIC	18 (12%)
	IV	4 (2%)
Histological grading	Grade 1	16 (10%)
	Grade 2	108 (70%)
	Grade 3	31 (20%)
Surgical margin	Negative	135 (87%)
	Positive	20 (13%)
SIRT1 expression	Low expression	78 (50%)
	Overexpression	77 (50%)

*AJCC* American Joint Committee on Cancer, *SIRT1* Sirtuin1

### Correlation between clinicopathologic parameters and SIRT1 expression

**Fig. 1:**
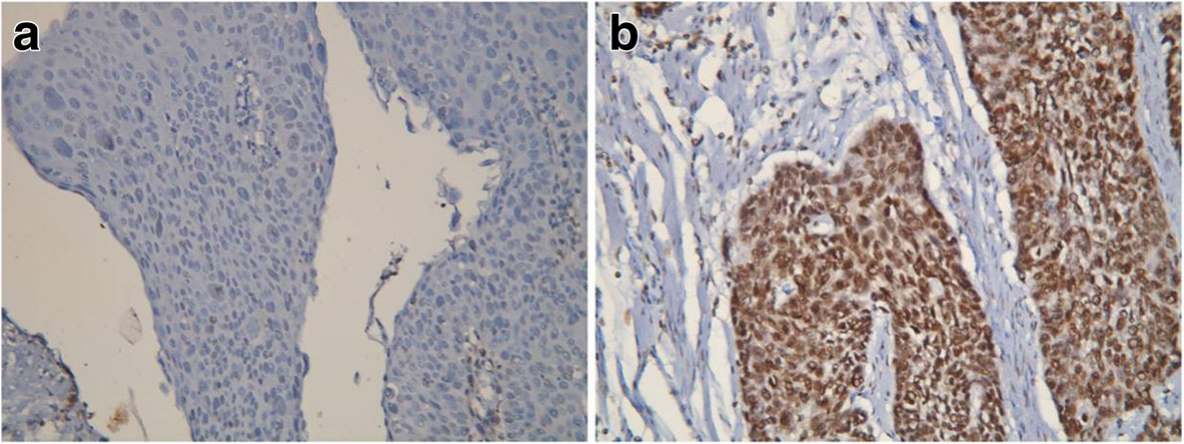
Immunohistochemical staining of SIRT1. **a** Low expression of SIRT1. **b** Overexpression of SIRT1. Original magnification × 200

Among the 155 patients, SIRT1 overexpression was identified in 77 (55%) patients (Fig. 1). There was no correlation between the clinicopathological factors and the IHC expression of SIRT1 (Table 2).

**Table 2:** Associations between SIRT1 expressions and clinicopathological parameters in 155 patients with esophageal squamous cell carcinoma receiving esophagectomy

Parameters		SIRT1 expression		
		Low	Over	*P* value
Age	<55y/o	32	37	0.38
	≧55y/o	46	40	
Sex	Male	75	75	1.00
	Female	3	2	
Primary tumor location	U + M	40	42	0.68
	L	38	35	
T classification	T1 + T2	45	34	0.092
	T3 + T4	33	43	
N classification	N0	55	51	0.57
	N1 + 2 + 3	23	26	
7^th^ AJCC Stage	I + II	57	55	0.82
	III + IV	21	22	
Histological grading	Grade 1 + 2	66	58	0.15
	Grade 3	12	19	
Surgical margin	Negative	75	60	0.38
	Positive	9	11	

*AJCC* American Joint Committee on Cancer, *SIRT1* Sirtuin1, *p-p70S6K* phosphorylated p70 ribosomal S6 protein kinase

### Survival analyses

**Fig. 2:**
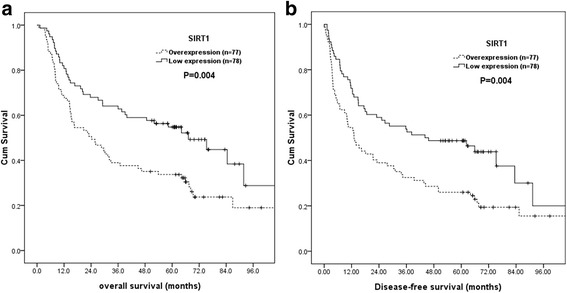
Kaplan–Meier curves according to SIRT1 status. **a** Overall survival according to SIRT1 status. **b** Disease-free survival according to SIRT1 status

Correlations of clinicopathologic parameters and SIRT1 with OS and DFS are shown in Table 3. Univariate analyses demonstrated that 7th AJCC stage III + IV (*P* < 0.001), T3 + 4 disease (*P* < 0.001), positive regional lymph node (*P* < 0.001), histological grading 3 (*P* = 0.001), positive surgical margin (*P* = 0.023), SIRT1 overexpression (*P* = 0.004, Fig. 2a) were associated with inferior OS. Additionally, 7th AJCC stage III + IV, T3 + 4 disease (*P* < 0.001), histological grading 3 (*P* = 0.002), positive regional lymph node (*P* < 0.001), positive surgical margin (*P* = 0.046) and SIRT1 overexpression (*P* = 0.004, Fig. 2b) were associated with inferior DFS. The 3-year OS and DFS rates were 63% and 54% in patients with low expression of SIRT1 and 39% and 33% in patients with overexpression of SIRT1, respectively. The 5-year OS and DFS rates were 55% and 49% in patients with low expression of SIRT1 and 34% and 26% in patients with overexpression of SIRT1, respectively.

**Table 3:** Results of univariate log-rank analysis of prognostic factors for overall survival and disease-free survival in 155 patients with esophageal squamous cell carcinoma receiving esophagectomy

Factors	No. of patients	Overall survival (OS)		Disease-free survival (DFS)	
		5-yr OS rate (%)	*P* value	5-yr DFS rate (%)	*P* value
Age					
< 55y/o	69	54%	0.19	48%	0.10
≧55y/o	86	37%		29%	
Location					
U + M	82	45%	0.84	35%	0.46
L	73	44%		40%	
T classification					
T1 + 2	79	64%	< 0.001^a^	52%	< 0.001^a^
T3 + 4	76	24%		22%	
N classification					
N0	106	56%	< 0.001^a^	47%	< 0.001^a^
N1 + 2 + 3	49	20%		16%	
7^th^ AJCC stage					
I + II	112	55%	< 0.001^a^	46%	< 0.001^a^
III + IV	43	16%		16%	
Histological grading					
Grade 1 + 2	124	51%	0.001^a^	43%	0.002^a^
Grade 3	31	19%		16%	
Surgical margin					
Negative	135	47%	0.023^a^	39%	0.046^a^
Positive	20	25%		25%	
SIRT1 expression					
Low expression	78	55%	0.004^a^	49%	0.004^a^
Overexpression	77	34%		26%	

*AJCC* American Joint Committee on Cancer, *SIRT1* Sirtuin1 ^a^Statistically significant

In multivariate comparisons, SIRT1 overexpression (*P* = 0.009, hazard ratio [HR], 1.776, 95% CI, 1.152–2.747) and T3 + 4 disease (*P* = 0.002, hazard ratio [HR], 2.250, 95% CI, 1.339–3.782) remained independently associated with inferior OS (Table 4). For DFS, SIRT1 overexpression (*P* = 0.017; HR, 1.642; 95% CI, 1.093–2.463), T3 + 4 disease (*P* = 0.005, hazard ratio [HR], 2.011, 95% CI, 1.240–3.261), positive regional lymph node (*P* = 0.035; HR, 1.967; 95% CI, 1.048–3.691) represented an independent adverse prognostic factor.

**Table 4:** Results of multivariate Cox regression analysis for overall survival and disease-free survival in155 patients with esophageal squamous cell carcinoma

Factors	Overall survival		Disease-free survival	
	HR (95% CI)	*P* value	HR (95% CI)	*P* value
T3 + 4	2.250 (1.339–3.782)	0.002^a^	2.011 (1.240–3.261)	0.005^a^
SIRT1 expression	1.776 (1.152–2.747)	0.009^a^	1.642 (1.093–2.463)	0.017^a^
N1 + 2 + 3	–	–	1.967 (1.048–3.691)	0.035^a^

*HR* hazard ratio, *95% CI* 95% confidence interval; ^a^Statistically significant

### The SIRT1 inhibitor suppressed cell proliferation of ESCC cell lines

**Fig. 3:**
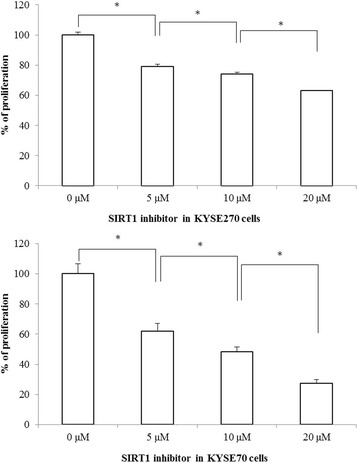
SIRT1 inhibitor displayed a proliferation-inhibitory effect in a dose-dependent manner in KYSE270 and KYSE70 esophageal squamous cell carcinoma cell lines. *Statistically significant difference in growth inhibition. Any *p* value < 0.05 was considered statistically significant. Columns, mean; bars, SD

Results on whether the SIRT1 inhibitor would suppress cell proliferation in ESCC cell line KYSE 270 and KYSE 70 show that the SIRT1 inhibitor, SIRT 1 inhibitor IV, (S)-35, displayed a dose-dependent, growth-inhibitory effect in both ESCC cell lines (Fig. 3a and b).

## Discussion

It is well known that advanced stage, higher histologic grade and residual disease after surgery lead to poor prognosis [1, 4, 5], and our study also showed the same result. In our study, we demonstrated that SIRT1 overexpression was an independent poor prognosticator for clinical outcome, and SIRT1 inhibitor suppressed cell proliferation of ESCC cell lines. Previous studies in several types of cancers [20–38] also showed SIRT1 overexpression was correlated with advanced stages or poor prognosis and inhibition of SIRT1 may suppress tumor progression. There are several possible mechanisms involved in SIRT1 mediated tumor progression. First, Liu et al. [18, 20] reported that SIRT1 can maintain silent chromatin via the deacetylation of histone proteins, and thus protect cells from apoptosis. Second, SIRT1 can repress tumor suppressor genes, such as p53, p27^kip1^, and FOXO family members, either by directly binding and deacetylating these non-histone proteins or by inducing heritable CpG island methylation at the gene promoter [18, 20, 30]. For example, previous studies [18, 20] showed that SIRT1 binds p53 and deacetylates its C-terminal Lys382, resulting in inhibition of p53 induction of cell cycle arrest and apoptosis in response to DNA damage and oxidative stress. Zhu et al. [30] reported that SIRT1 is an important regulator of p27^kip1^ and SIRT inhibition induces senescence and antigrowth potential in lung cancer. Third, Byles et al. [27] also found that SIRT1 can enhance metastatic potential by inducing epithelial-mesenchymal transition in prostate cancer. Fourth, previous studies [29, 31] showed that SIRT1 is involved in chemotherapy resistance. Liang et al. [29] suggest that reduced glucose use and altered mitochondrial metabolism mediated by SIRT1 may contribute to cisplatin resistance. Taken together, the results from our study, together with previous findings, suggest that SIRT1 is not only an adverse prognosticator but also a potential novel therapeutic target.

Despite advance in perioperative management and surgical techniques, the prognosis of patients with ESCC remains poor [1, 2]. Even after radical surgery, patients still develop recurrences and metastases. Over the past decades, several post-operative adjuvant therapy clinical trials were performed to improve unsatisfactory cure rate achieved with surgery alone. Hence, identifying patients at high risk for recurrence who may benefit from post-operative adjuvant therapy is principal. In our study, overexpression of SIRT1 was highly representative of biological aggressiveness and independently associated with worse disease-free survival. The 5-year disease-free survival rate was only 26% in patients with SIRT1 overexpression, indicating that SIRT1 status may be used to select some patients for adjuvant therapy after esophagectomy.

## Conclusions

Higher expression of SIRT1 is an independent prognosticator for patients with ESCC. The SIRT1 inhibitor suppressed cell proliferation of ESCC in vitro. Our findings suggest that Sirtuin inhibitors may be a potential therapy in ESCC patients with SIRT1 overexpression. There were two limitations in our study. First, the patient number was small and was retrospectively analyzed. Second, the effect of SIRT1 inhibitor was analyzed in cell line. The results need further studies to confirm our findings.

## Abbreviations

AJCC: American Joint Committee on Cancer
DAB: Diaminobenzidine tetrahydrochloride
DFS: Disease-free survival
DNA: Deoxyribonucleic acid
ECACC: European collection of authenticated cell cultures
EDTA: Ethylene diamine tetraacetie acid
ESCC: Esophageal squamous cell carcinoma
HDAC: Histone deacetylases
HR: Hazard ratio
MTT: diphenyltetrazolium bromide
NDA: Nicotinamide adenine dinucleotide
OS: Overall survival
PBS: Phosphate buffered saline
pH: potential of hydrogen
SIRT1: Sirtuin 1
TNM: Tumor, nodes and metastasis
